# Fibrin Clot Formation under Oxidative Stress Conditions

**DOI:** 10.3390/antiox10060923

**Published:** 2021-06-07

**Authors:** Jirina Kaufmanova, Jana Stikarova, Alzbeta Hlavackova, Leona Chrastinova, Martin Maly, Jiri Suttnar, Jan Evangelista Dyr

**Affiliations:** 1 Department of Biochemistry and Microbiology, University of Chemistry and Technology Prague, Technicka 5, 166 28 Prague, Czech Republic; Jirina.Kaufmanova@vscht.cz; 2 Department of Biochemistry, Institute of Hematology and Blood Transfusion, U Nemocnice 1, 120 00 Prague, Czech Republic; Alzbeta.Hlavackova@uhkt.cz (A.H.); Leona.Chrastinova@uhkt.cz (L.C.); Jiri.Suttnar@uhkt.cz (J.S.); jan.dyr@uhkt.cz (J.E.D.); 3 Department of Medicine, First Faculty of Medicine, Charles University in Prague and Military University Hospital, U Vojenske Nemocnice 1200, 169 02 Prague, Czech Republic; martin.maly@uvn.cz

**Keywords:** fibrinogen, acute coronary syndrome, stroke, artery stenosis, oxidative stress

## Abstract

During coagulation, the soluble fibrinogen is converted into insoluble fibrin. Fibrinogen is a multifunctional plasma protein, which is essential for hemostasis. Various oxidative posttranslational modifications influence fibrinogen structure as well as interactions between various partners in the coagulation process. The aim was to examine the effects of oxidative stress conditions on fibrin clot formation in arterial atherothrombotic disorders. We studied the changes in in vitro fibrin network formation in three groups of patients—with acute coronary syndrome (ACS), with significant carotid artery stenosis (SCAS), and with acute ischemic stroke (AIS), as well as a control group. The level of oxidative stress marker malondialdehyde measured by LC-MS/MS was higher in SCAS and AIS patients compared with controls. Turbidic methods revealed a higher final optical density and a prolonged lysis time in the clots of these patients. Electron microscopy was used to visualize changes in the in vitro-formed fibrin network. Fibers from patients with AIS were significantly thicker in comparison with control and ACS fibers. The number of fibrin fibers in patients with AIS was significantly lower in comparison with ACS and control groups. Thus, oxidative stress-mediated changes in fibrin clot formation, structure and dissolution may affect the effectiveness of thrombolytic therapy.

## 1. Introduction

During coagulation, the soluble fibrinogen is converted into insoluble fibrin, which, together with platelets, produces the hemostatic clot. Fibrinogen is synthesized in hepatocytes and secreted into the blood, where it circulates with a half-life of 3 days [1] and an average concentration of about 2.5 g/L [2]. Fibrinogen is a 340 kDa glycoprotein, consisting of three non-identical peptide chains, Aα, Bβ, and γ, consisting of 610, 461 and 411 amino acid residues with molecular weights of 67.5, 55 and 46.5 kD, respectively [3]. The chains are connected together by 29 disulfide bonds [4,5]). The process of coagulation is initiated by a serine protease thrombin. Fibrin is covalently crosslinked by the transglutaminase factor XIII (FXIII), which provides stability, elasticity and resistance to the fibrinolysis of the clot [1,4]. FXIII also links α2-antiplasmin and plasminogen activator inhibitors to fibrin to ensure clot resistance to enzymatic degradation [6]. Fibrinogen and fibrin specifically bind a variety of proteins, including albumin, apolipoproteins, complement C3, ferritin, fibronectin, haptoglobin, myosin, plasminogen, vascular endothelial growth factor, von Willebrand factor and some others [7]. The structure of a fibrin clot and its resistance to mechanical deformation and/or enzymatic degradation is influenced by environmental and genetic factors and depends on the fibers’ individual properties [6,8,9].

Proteins are sensitive biomarkers of human (patho)physiological conditions associated with oxidative stress, and fibrinogen seems to be the most vulnerable target for oxidant attack [3,10,11]. Oxidative stress occurs in the human body when a high amount of reactive oxygen and nitrogen species (ROS, RNS) are produced by immune cells, pro-oxidant enzymes, and during oxygen and nitrogen metabolism which are not sufficiently detoxified by antioxidants [12,13]. ROS and RNS can be also produced as a consequence of exogenous effects, for example, radiation from UV light or smoking [14]. ROS/RNS play a role as messengers in various signaling pathways at physiological concentrations. Under unbalanced high concentrations of ROS/RNS, cell and tissue damage caused by modifications of proteins, nucleic acids, lipids, etc., occurs [13]. The vulnerability of the protein to modification by ROS/RNS is associated with its primary and three-dimensional structure [15]. The alteration of protein structures by oxidants may result in partial or complete loss of protein function [10]. Posttranslational oxidative modifications of fibrinogen molecules causes the alternative assembly of fibrin into so-called thrombogenic fibrin with an abnormal structure and properties and a reduced strength and elasticity [3,16]. Clot formation, clot characteristics and susceptibility to fibrinolysis is also affected [12].

The aim of this work was to observe functional and structural changes of fibrin formed in vitro from the plasma of patients with acute coronary syndrome (ACS), acute ischemic stroke (AIS), significant carotid artery stenosis (SCAS) and the control group (control).

## 2. Materials and Methods

### 2.1. Subjects and Ethics Statement

Blood samples were collected from 4 patient groups (Table 1): (A) from acute coronary syndrome (ACS) patients indicated for percutaneous coronary intervention (PCI), where blood samples were collected from an arterial sheath inserted into a radial or femoral artery, depending on the approach for intervention; (B) from patients with significant carotid artery stenosis (SCAS) who were diagnosed using ultrasound carotid artery stenosis screening with a severity of the carotid artery stenosis 81 ± 12% (mean ± SD) where samples were drawn by venipuncture; (C) from patients with acute ischemic stroke (AIS) indicated for endovascular treatment with large vessel occlusion, where sampling was carried out just before intervention in the same manner as in the ACS group, and (D) from a control group, where blood samples were obtained from patients undergoing coronary angiography due to non-coronary principal diagnoses and found with nonsignificant atherosclerotic changes on coronary arteries (valve disease, cardiomyopathies, heart failure, atypical chest pain). Samples from these patients were drawn and processed in the same way as samples from patients with ACS. All the samples were obtained and analyzed in accordance with the Ethical Committee regulations of the Military University Hospital, Prague, Czech Republic (108/11-49/2017) and the Ethical Committee regulations of the Institute of Hematology and Blood Transfusion, Prague, Czech Republic (EK 9/AZV CR/06/2017). Prior to enrollment in the study, written informed consent was obtained from each subject. All data were analyzed anonymously. The study was carried out in accordance with the International Guidelines and the Declaration of Helsinki. Blood samples were collected to 1 mL of 3.8% trisodium citrate to a final volume of 9 mL. The samples were centrifuged at 1400× *g* at 25 °C for 10 min to obtain platelet-poor plasma.

### 2.2. Materials

All chemicals were obtained from Sigma-Aldrich (Prague, Czech Republic) unless otherwise specified. Chromatographic solvents were from Merck (Prague, Czech Republic). All of the reagents employed were of analytical grade or higher purity, and all aqueous solutions were prepared using HPLC-grade water.

### 2.3. Fibrinogen Immunoassay

Fibrinogen concentration was determined using the Fibrinogen Immunoturbidimetric Assay (KAI-135; Kamiya Biomedical Company, Seattle, WA, USA). The plasma samples were diluted with saline solution in a ratio of 1:20 and incubated at 37 °C with R1 buffer (Tris(hydroxymethyl)aminomethane, 100 mM) for 5 min. After the addition of R2 buffer (anti-human fibrinogen goat antiserum, 30%), samples were again incubated at 37 °C for 5 min, and the reaction was determined by measuring the turbidity spectrum from 350 to 700 nm using the Synergy HT spectrophotometer (Bio-tek Instruments, Winooski, VT, USA). Samples were analyzed in duplicates.

### 2.4. Determination of Malondialdehyde Concentration in Plasma

The total malondialdehyde concentration in the plasma was determined after alkaline hydrolysis with sodium hydroxide using the liquid chromatography–tandem mass spectrometry (LC-MS/MS) method according to Bechynska et al. [17].

### 2.5. Fibrin Polymerization Curve Measurement and Fibrinolysis

The plasma samples were diluted with TRIS buffer pH 7.4 in a ratio of 1:3 and incubated at 37 °C with thrombin (9 NIH U/mL, final concentration) and CaCl_2_ (8 mM, final concentration). Thrombin-catalyzed fibrin polymerization was immediately measured, reading turbidity at 350 nm in 20 s intervals for 40 min using an ELISA reader Synergy HT spectrophotometer (Bio-tek Instruments, Winooski, VT, USA). Samples were analyzed in duplicates.

Clot degradation was measured in diluted plasma (1:3 with a TRIS buffer pH 7.4) from the patient or the control after the addition of thrombin (12 NIH U/mL, final concentration), plasminogen (0.15 NIH U/mL, final concentration), tPA (0.3 µg/mL, final concentration), and CaCl_2_ (8 mM, final concentration). A reaction was detected by measuring turbidity at 350 nm every 20 s for 40 min, using an ELISA reader Synergy HT spectrophotometer (Bio-tek Instruments, Winooski, VT, USA), and 2 replicate measurements were performed for each sample. 

### 2.6. Scanning Electron Microscopy

The fibrin network architecture was studied with scanning electron microscopy (Mira 3 LMH, Tescan Orsay Holding, a.s., Brno, Czech Republic). Fibrinogen from the patient and control samples was mixed with Ca^2+^ (8 mM, final concentration) and thrombin (final concentration 2 NIH U/mL) and incubated at room temperature for 3 h. The networks were then fixated with 4% formaldehyde overnight. The fixed samples were washed with PBS and water and subsequently dehydrated with a series of water–ethanol solutions with increasing ethanol concentration (30%, 50%, 70%, 80%, 90%, 95% and 2× 100%). Finally, the samples were dried using the CO_2_ critical point method (Leica EM CPD300) and coated with 4 nm thick gold by sputtering (Leica EM ACE600). The fibrin networks were studied with an SEM, and the samples were methodically viewed to establish the consistency of the ultrastructure. Images were evaluated using ImageJ data analysis software (http://rsbweb.nih.gov/ij/; accessed on 13 May 2021). Fiber thickness and the average number of fibrin fibers per 1 micrometer square of fibrin clot were determined by analyzing ten different images captured from two independently prepared samples. Fiber thickness was determined from 100 values (10 measurements per image). The average number of fibers per 1 micrometer square was determined from 10 images per sample. 

### 2.7. Statistical Analysis

Statistical analyses were performed using R software [18] (and GraphPad Prism (version 8.1.2 for Windows, GraphPad Software, San Diego, CA, USA, www.graphpad.com, accessed on 13 May 2021). The differences among groups were evaluated by the Kruskal–Wallis test at *p* < 0.05. When the Kruskal–Wallis test was statistically significant, a post hoc Dunn test was performed. ANOVA was used to examine the differences in MDA concentration across all subgroups of patients at *p* < 0.05. When ANOVA was statistically significant, a post hoc Dunn test was performed. Chi-square was used for the comparison of patients’ groups. For the assessment of correlation between various data, Pearson correlation tests were used with pairwise two-sided *p*-values.

## 3. Results

The changes in fibrin network formation were studied in patients with ACS, SCAS, AIS, and a control group. The characteristics of patient groups are listed in Table 1.

When the Kruskal–Wallis test was statistically significant, a post hoc Dunn test was performed. The post hoc Dunn test revealed significant differences in age between ACS and AIS (*p* = 0.009), in dyslipidemia between SCAS and ACS (*p* = 0.006).

### 3.1. Fibrinogen Plasma Concentration

Fibrinogen concentration in the plasma of patients’ samples was determined by immunoassay. There were no significant differences among the groups (Table 2). 

### 3.2. Malondialdehyde Plasma Concentration

The concentration of malondialdehyde in the plasma was measured (Table 3). A significantly higher MDA concentration was detected in the SCAS and AIS groups compared to the control group (*p* = 0.004, *p* = 0.003, respectively) as well as to the ACS group (*p* = 0.014, *p* = 0.021, respectively). The MDA levels of ACS patients were not significantly higher in comparison with the control samples.

### 3.3. Fibrin Polymerization Curve Measurement and Fibrinolysis

Fibrin formation in the plasma was monitored by turbidimetry at 350 nm for 40 min. 

**Figure 1:**
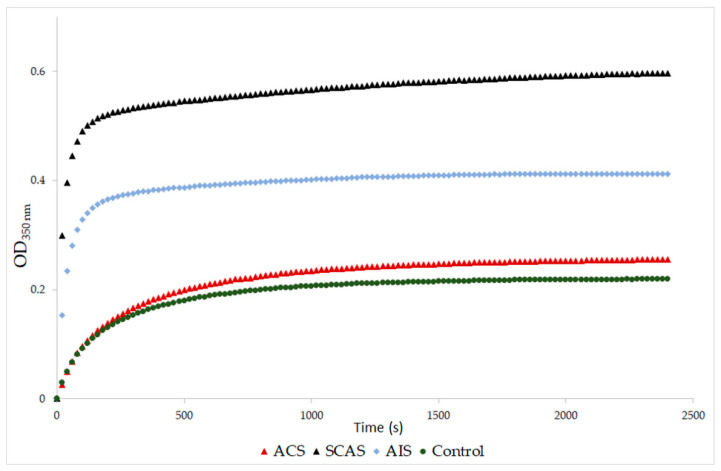
Representative curves of thrombin-catalyzed fibrin polymerization. ACS: patients with acute coronary syndrome; SCAS: patients with significant carotid artery stenosis; AIS: patients with acute ischemic stroke. Fibrin clot formation was monitored at 350 nm after addition of thrombin (9 NIH U/mL) and CaCl_2_ (8 mM).

**Figure 2:**
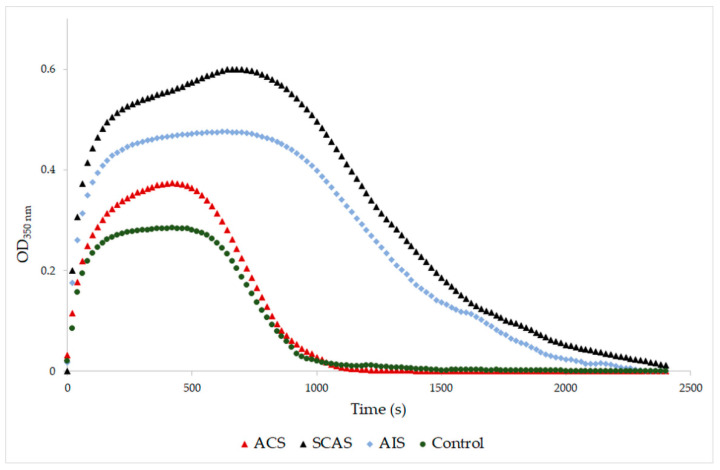
Representative curves of thrombin-catalyzed fibrin polymerization and fibrinolysis. ACS: patients with acute coronary syndrome; SCAS: patients with significant carotid artery stenosis; AIS: patients with acute ischemic stroke; control: patients with normal angiogram. Clot degradation was measured at 350 nm after addition of thrombin (12 NIH U/mL), plasminogen (0.15 NIH U/mL), tPA (0.3 µg/mL) and CaCl_2_ (8 mM).

A significantly higher final optical density was found in samples from patients with SCAS and with AIS compared to the control samples (*p* = 0.001, *p* = 0.029, respectively; Table 4). The same results were obtained when the SCAS and AIS groups were compared to the ACS group (*p* = 0.001, *p* = 0.029, respectively). The final optical density of samples from patients with ACS was similar to the control samples (Figure 1). Samples from patients with SCAS or AIS had significantly prolonged lysis time compared to those with ACS (*p* < 0.001, *p* = 0.009, respectively; Table 4, Figure 2). The differences between the control group and both the SCAS and AIS groups were not significant, but lysis times were noticeably longer in both cases.

### 3.4. Scanning Electron Microscopy

**Figure 3:**
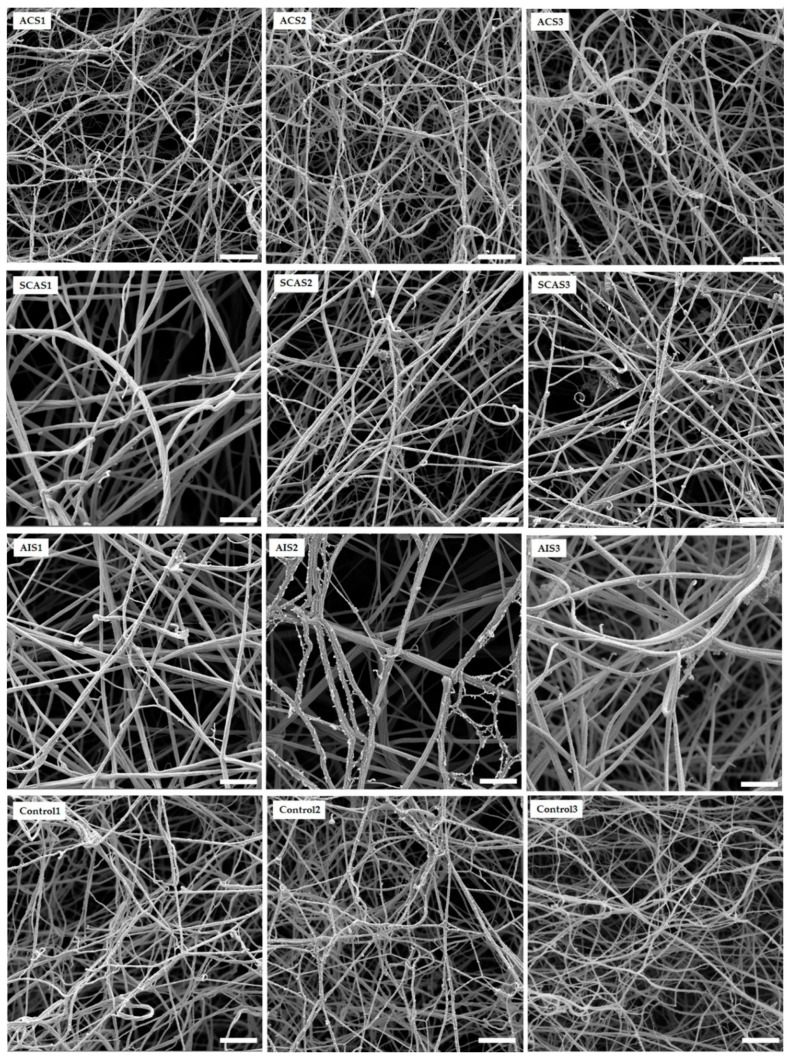
Representative SEM images of in vitro fibrin clots formed by patients and control fibrinogens. ACS: patients with acute coronary syndrome; SCAS: patients with significant carotid artery stenosis; AIS: patients with acute ischemic stroke; control: patients with normal angiogram. The scale bar is 2 µm.

The architecture of the networks generated by the fibrin in vitro was examined using a scanning electron microscope (SEM; Figure 3). The samples were methodically viewed to establish the consistency of the ultrastructure. Ten different images captured from two independently prepared samples were used for the analysis of fiber thickness and average number of fibrin fibers. Significantly thicker fibrin fibers were found in samples from patients with AIS as compared with controls and ACS (*p* < 0.001, *p* < 0.001, respectively). Additionally, in AIS patients, we found significantly lower numbers of fibrin fibers in comparison with controls and ACS (*p* < 0.001, *p* = 0.023, respectively) (Table 5). 

## 4. Discussion

It is known that an increased risk of bleeding or thrombosis is associated with abnormal thrombin generation and the production of clots with an altered fibrin structure [19]. Recent studies showed that the modification of fibrinogen molecules affects hemostasis by producing changes in the formation and architecture of the fibrin network and changing how fibrin/fibrinogen interacts with platelets, endothelium, and other cells via cell-membrane fibrin/fibrinogen receptors [4,5,20]. Parameters that confirm the changed coagulation include an activated intrinsic pathway, prothrombin consumption, increased thrombin activity, elevated fibrinogen levels, augmented platelet counts and increased thrombin activity on platelet surfaces [21,22,23]. In cardiovascular diseases, the prothrombotic fibrin clot phenotype could be found [6].

This study focused on altered clot formation in patients with acute coronary syndrome, patients with significant carotid artery stenosis, and patients with acute ischemic stroke. An objective of this study was to examine clot formation, structure and dissolution; citrated plasma was used in all experiments. Turbidimetry was applied to monitor fibrin formation and dissolution, and an SEM was used for the visualization of fibrin clot structures. 

In this study, plasma fibrin clots of patients with SCAS and AIS were composed of thicker and fewer fibers compared to control samples. Clots of these patients had a higher final optical density and a prolonged lysis time. The MDA levels in the plasma were also significantly higher in these patient groups. The characteristic of clots formed from the plasma of ACS patients were similar to clots from the plasma of the control group.

Fibrinogen is known as the main determinant of fibrin clot structure and physiologically undergoes many posttranslational modifications. The literary data of the impact of fibrinogen concentration to fibrin formation are not straightforward. [6,9]. Undas et al. proposed that increased levels of fibrinogen results in faster activation rate, a denser and tighter fibrin network, as well as the formation of thicker fibers [9]. On the other hand, Zabczyk et al. stated that at low concentrations of fibrinogen, clots are composed of thicker fibers, while clots formed at higher concentrations of fibrinogen are composed of thinner fibers [6]. In this study, immunoassay was employed for the determination of the total fibrinogen concentration. No significant differences in the concentrations of fibrinogen were found among the ACS, SCAS, AIS and control groups. It should be considered that the levels of total fibrinogen determined by immunoassay may be different from its functional levels. As has been described, post-translational modifications of fibrinogen change its function. The highest MDA levels were observed in SCAS and AIS patients, with clearly different fibrin clot formation and architecture. It might be, in accordance with Zabczyk et al. [6], that a low concentration of functionally capable fibrinogen is accountable for fibrin architecture.

Functional changes of fibrinogen could be caused by posttranslational modifications of the fibrinogen molecule. The oxidative modification of fibrinogen and its effect on clot formation and fibrinolysis is well described [6,9,24,25,26,27]. The degree of impairment of fibrinogen is linked to the duration and strength of oxidative stress [5]. The groups of patients most affected by oxidative stress, which was assessed using MDA levels, were those with SCAS and AIS with distinguished changes in clot formation and lysis in comparison with the control group.

Aging is intertwined with oxidative stress as a cause and consequence of many pathological conditions. Glycosylation was reported as a main modification of fibrinogen in the aging process, primarily affecting clotting time [28]. In our study, the ACS group was significantly different, with most specimens being younger than in other groups. However, this group of patients had the most similar results to the control group, even though the age difference was significant. 

The concentration of fibrinogen and alterations in fibrin clot properties could also be mediated by statins, which are used in the management of hyperlipidemia. Statins are associated with the modulation of PAI-1 and tPA activity, influence fibrinogen concentration, and have an impact on fibrinolysis [29,30,31]. Statins could increase the permeability of clots and thus enhance fibrinolysis [30]. In this study, statins were mainly prescribed in the SCAS patients. However, this group also had prolonged fibrinolysis. Statins are linked with an increase in maximum absorbance during turbidimetric measurement [30]. Our data suggested a weak positive correlation between taking statins and peak turbidity (r = 0.32; *p* = 0.06).

Fibrinogen function could be affected by acetylation linked with acetylsalicylic acid (ASA). ASA is linked with shorter fibrinolysis, thicker fibers and lower network density [32,33,34,35]. In this study, no significant differences were found in taking ASA. There were no correlations between taking ASA and fiber diameter, network density or fibrinolysis (r = 0.063, *p* = 0.713; r = −0.019, *p* = 0.267; r = 0.004, *p* = 0.549; reps.).

Hyperglycemia, as well, might impair clot structure and susceptibility to lysis [24]. No significant differences were observed among the groups of patients and the control group in glycated hemoglobin (Table 1). 

Glucose-lowering agents are linked to positive effects in hemostasis. Metformin and its derivates are known for a profibrinolytic effect, a beneficial effect on vascular endothelium and the prolongation of platelet thrombus formation [36]. Acarbose has a similar [37]. In this study, no correlations were found between HbA1c and fibrinolysis or between HbA1c and maximal turbidity (r = 0.020, *p* = 0.908; r = 0.006, *p* = 0.970; resp.). Additionally, no correlations were found between DM and fibrinolysis or between DM and maximal turbidity (r = 0.113, *p* = 0.486; r = 0.242, *p* = 0.133; resp.).

Smoking is one of exogenous factors that affect fibrin clot formation and characteristics. Barua et al. observed the effect of cigarette smoke exposure to the architecture of fibrin clots. They described denser clots with thinner fibers with higher maximal turbidity in a group of smokers, especially after smoking [38]. In our study, no correlations were found between smoking and fiber diameter, network density, maximal turbidity or fibrinolysis (r = 0.061, *p* = 0.720; r = 0.006, *p* = 0.974; r = 0.119, *p* = 0.471; r = −0.820, *p* = 0.621; reps.). 

Acute coronary syndrome, associated mostly with atherosclerotic plaque rupture, results in a blood flow blockade in an infarct-related artery area. Clots formed from the plasma of ACS patients demonstrated thinner fibers compared to those with AIS and SCAS and had the highest density, albeit nonsignificant, among the groups. The decreased clot permeability in patients with acute myocardial infarction could be related to the degree of oxidative stress and inflammation [24]. Hoffman [39], Martinez et al. [1] and Weigandt et al. [40] describe different oxidative modifications of fibrinogen molecules, which were associated with various changes in clot structure and function. Increased oxidative stress, platelet activation, and thrombin generation play a key role in the pathogenesis of acute coronary syndrome. The MDA level of ACS patients was slightly higher compared to the controls, but significantly lower compared to SCAS and AIS groups. Mahreen et al. proposed that serum MDA levels can be increased because of tissue damage caused by myocardial infarction, resulting in an increased rate of production of free radicals [41]. Aznara et al. published a study where the maximal MDA level had been observed between 6 and 8 days after infarction [42]. This phenomenon might be explained by reperfusion in the ischemic region. This sudden resumption of blood flow in ischemic tissue causes the higher production of ROS and the further damage of tissue [43]. ACS patients were probably in the stage of their lower level of MDA concentration, which might increase in the days after infarction. 

A major percentage of all acute strokes result from precerebral or cerebral artery occlusions. Thrombi found that the cerebral arteries of patients with acute ischemic stroke are usually composed of fibrin and platelet deposits, with erythrocyte components [44]. Abnormal plasma fibrin clot properties have been described in patients who survived ischemic stroke. Undas et al. [45] reported these prothrombotic clot abnormalities in patients with cryptogenic stroke. They found out that in vitro generated clots of these patients are more compact, with an increased fiber diameter and density. In this study, AIS patients were found also to have an increased fiber diameter but with lower density compared to the control group. The fibrinolysis of the AIS patients was prolonged (Figure 2). The MDA level of AIS patients was significantly elevated compared to the control group, which agrees with Elsayed et al. [46] and Polidori et al. [47], who published high levels of MDA in AIS patients. Sovová et al. [20] reported a significantly lower number of fibrin fibers with an increased fiber diameter in the fibrinogen samples modified by MDA. In this study, the groups with high levels of MDA also had an increased fiber diameter together with a decreased number of fibrin fibers. Our data suggested a medium positive correlation between MDA concentration and fibers thickness (r = 0.578; *p* < 0.001) and a medium negative correlation between MDA concentration and density of fibrin network (r = −0.439; *p* = 0.008).

Patients with SCAS are rarely studied. To our knowledge, we present the first study of clots prepared in vitro from citrated plasma obtained from SCAS patients. The concentration of fibrinogen was similar to the control group, but MDA was significantly elevated. The SCAS group of patients had the longest fibrinolysis as well as the highest peak turbidity. Their fibrin clots were characterized by thicker and fewer fibers in comparison to the control group. Hajšl et al. [48] presented data of oxidative stress in SCAS and AIS patients. In our study, MDA did not differ significantly between SCAS and AIS groups, unlike in Hajšl et al.’s, where a significant difference was found. This could be caused by a lower number of probands in each group. 

Our study has some limitations. One is its small number of participants. Another is that our experimental approach did not allow for the analysis of the effect of blood cells and platelets on fibrin clot structure/function. The interaction of fibrin(ogen) with platelets might also be affected as well as the stability of formed clot under blood flow. However, this study focused only on fibrin clot formation, structure and dissolution. 

In vivo clot formation may be also influenced by metabolites, products of (patho)physiological processes. Enzymatic activity may lead to the production of RONS and also may have an antioxidant effect; both outcomes can affect fibrinogen or the dynamics of coagulation. An example of ambivalent enzymatic activity could be the heme oxygenase family, which can produce ferrous iron and carbon monoxide, linked to changes of fibrin structure and coagulation kinetics [49,50], and biliverdin [51]. Heme oxygenase may have a protective role in coronary artery diseases [52,53], ischemic [54] and hemorrhagic [55] stroke, in terms of the degradation of heme. Enzymatic activity and metabolites were not analyzed for groups or for individual patients. 

The plasmatic level of MDA was used as the only marker of oxidative stress. Nevertheless, MDA concentration determined by LC-MS/MS is a reliable marker of oxidative stress.

In vitro clot formation and architecture was significantly different in SCAS and AIS patients compared to the control group and patients with ACS. The clots were less dense, with thicker fibers. The SCAS group showed the highest maximal absorbance among the observed groups, and their fibrinolytic time was the longest one. These observations could be associated with oxidative stress, as evidenced by higher MDA levels. Changes of the fibrinogen molecule mediated by oxidative stress reagents not only influence modified residuum but might also alter fibrinogen’s secondary structure [20]. 

## 5. Conclusions

In conclusion, oxidative modifications occurring in patients with ACS, SCAS and AIS alter the fibrinogen functionality that determines the efficiency of coagulation and fibrinolysis. Although the studied diseases have the same pathophysiological basis, the architecture and properties of the fibrin network are unique to each individual disease. It may be interesting to monitor fibrin networks for individual treatment-related thrombotic diseases.

## Figures and Tables

**Table 1:** Patients’ characteristics.

Patient Group Descriptions	ACS	SCAS	AIS	Control	*p*-Value
	(*n* = 10)	(*n* = 10)	(*n* = 10)	(*n* = 10)	
Demographic and risk factors					
Age	60.0 (47.0–70.0)	69.5 (65.0–79.0)	73.5 (69.0–86.0)	69.0 (51.0–77.0)	0.02 ^1^
Sex female/male	3/7	4/6	3/7	7/3	0.22 ^2^
Diabetes mellitus	3	7	4	2	0.12 ^2^
Active smoking	5	4	1	2	0.19 ^2^
Arterial hypertension	6	7	8	8	0.50 ^2^
Dyslipidemia	2	9	3	6	0.01 ^2^
BMI (mean ± SD)	28.07 ± 4.10	26.85 ± 4.06	30.26 ± 5.24	30.57 ± 4.49	0.21 ^1^
Pre-admission antithrombotic treatment					
Acetylsalicylic acid	2	5	3	4	
Clopidogrel	0	5	1	0	
Fraxiparine	0	1	0	0	
DOACs	0	0	0	2	
Warfarin	0	0	0	1	
Pre-admission dyslipidemic treatment					
Statins	2	9	3	6	
Fenofibrate	0	0	0	1	
Previous coronary events					
Percutaneous coronary intervention	1	0	1	0	
Myocardial infarction	2	1	2	0	
Coronary artery bypass graft	0	0	2	0	
Ischemic stroke	0	5	10	0	
Peripheral artery disease	0	4	2	0	
Clinical presentation					
Triacylglycerols (mM)	0.95 (0.76–1.31)	1.49 (1.00–1.72)	1.10 (0.61–2.18)	0.99 (0.64–1.13)	0.08 ^1^
Total cholesterol (mM)	4.50 (3.87–5.44)	4.52 (3.19–4.95)	4.21 (2.32–5.40)	3.85 (3.48–5.15)	0.92 ^1^
High density lipoproteins (mM)	1.03 (0.84–1.42)	1.36 (0.96–2.07)	0.96 (0.84–1.41)	1.44 (1.03–1.90)	0.08 ^1^
Low density lipoproteins (mM)	3.04 (2.01–3.64)	2.00 (1.53–2.82)	2.58 (0.92–3.65)	2.13 (1.62–3.06)	0.31 ^1^
Glycosylated hemoglobin (mmol/mol)	40.5 (36.0–96.0)	50.5 (40.0–69.0)	39.0 (33.0–57.0)	39.0 (37.0–54.0)	0.13 ^1^

ACS: acute coronary syndrome; SCAS: significant carotid artery stenosis; AIS: acute ischemic stroke; BMI: body mass index; SD: standard deviation; NA: not available. Values are given as mean ± SD, median 5–95th percentiles in parentheses. ^1^ Kruskal–Wallis test, ^2^ Chi-square.

**Table 2:** Fibrinogen concentration.

	ACS	SCAS	AIS	Control	*p*-Value
Fbg (mg/mL)	5.11 (3.98–6.72)	4.513 (4.11–5.06)	3.49 (2.91–5.23)	4.47 (3.33–6.37)	0.28

The values are presented as median with 5–95th percentiles in parentheses. Kruskal–Wallis test was used to examine the differences across all subgroups of patients. Abbreviations: ACS: patients with acute coronary syndrome; SCAS: patients with significant carotid artery stenosis; AIS: patients with acute ischemic stroke.

**Table 3:** Malondialdehyde plasma concentration.

	ACS	SCAS	AIS	Control	*p*-Value
MDA (µM)	3.07 ± 1.28	4.55 ± 0.86	4.48 ± 1.21	2.72 ± 0.51	<0.001

The values are presented as averages ± SD. ANOVA was used to examine the differences across all subgroups of patients at *p* < 0.05. When ANOVA was statistically significant, a post hoc Dunn test was performed. Abbreviations: ACS: patients with acute coronary syndrome; SCAS: patients with significant carotid artery stenosis; AIS: patients with acute ischemic stroke.

**Table 4:** Final optical density and lysis time.

	ACS	SCAS	AIS	Control	*p*-Value
Peak turbidity	0.23 (0.09–0.47)	0.59 (0.41–0.83)	0.50 (0.35–0.60)	0.21 (0.13–0.41)	<0.001
Lysis time (min)	35.5 (30.3–38.7)	40.0 (38.0–40.0)	39.7 (34.7–40.0)	37.3 (27.3–39.3)	<0.001

The values are presented as median with 5–95th percentiles in parentheses. Kruskal–Wallis test was used to examine the differences across all subgroups of patients at *p* < 0.05. When Kruskal–Wallis test was statistically significant, a post hoc Dunn test was performed. Abbreviations: ACS: patients with acute coronary syndrome; SCAS: patients with significant carotid artery stenosis; AIS: patients with acute ischemic stroke.

**Table 5:** Fiber thickness and average number of fibrin fibers per 1 µm^2^ of fibrin clot.

	ACS	SCAS	AIS	Control	*p*-Value
Fiber thickness (µm)	0.117 (0.113–0.120)	0.135 (0.131–0.140)	0.1645 (0.156–0.172)	0.116 (0.112–0.121)	<0.001
Average no. of fibers per field (1 µm^2^)	7.79 (7.52–8.01)	5.97 (5.68–6.22)	5.11 (4.67–5.33)	6.67 (6.50–6.89)	<0.010

The values are presented as median with 5–95th percentiles in parentheses. Kruskal–Wallis test was used to examine the differences across all subgroups of patients at *p* < 0.05. When Kruskal–Wallis test was statistically significant, a post hoc Dunn test was performed. Abbreviations: ACS: patients with acute coronary syndrome; SCAS: patients with significant carotid artery stenosis; AIS: patients with acute ischemic stroke.

## Data Availability

The data presented in this study are available on request from the corresponding author.
